# Arbuscular mycorrhizal root colonization depends on the spatial distribution of the host plants

**DOI:** 10.1007/s00572-022-01087-0

**Published:** 2022-07-06

**Authors:** Leonie Grünfeld, Georgios Skias, Matthias C. Rillig, Stavros D. Veresoglou

**Affiliations:** 1 Institut Für Biologie, Freie Universität Berlin, Altensteinstr. 6, 14195 Berlin, Germany; 2 Berlin-Brandenburg Institute of Advanced Biodiversity Research, 14195 Berlin, Germany; 3 State Key Laboratory of Biocontrol, School of Ecology, Sun Yat-Sen University, Shenzhen, 518107 China

**Keywords:** Arbuscular mycorrhizal fungi, Fungal dispersal, Glomeromycota, Habitat fragmentation, Micro-landscapes

## Abstract

Despite their ubiquity in terrestrial ecosystems, arbuscular mycorrhizal fungi (AMF) experience dispersion constraints and thus depend on the spatial distribution of the plant hosts. Our understanding of fungal-plant interactions with respect to their spatial distributions and implications for the functioning of the symbiosis remain limited. We here manipulated the location of habitat patches of *Medicago lupulina* in two experiments to explore the responses of AMF root colonization and extraradical hyphae. We tested the specific hypothesis that AMF-plant habitats high in connectance would stimulate root colonization and induce denser functional root colonization (colonization rate of arbuscules plus coils) because of higher propagule availability between nearby host plant patches (*experiment 1*). In *experiment 2*, we anticipated similar responses in mixed habitats of different soil fertility, namely phosphorus-fertilized or unfertilized soil, and anticipated a higher density of extraradical hyphae in the soil connecting the habitats with increased functional root colonization. In agreement with our hypothesis, we found the highest total and functional root colonization in unfragmented micro-landscapes, describing landscapes that occur within a spatial scale of a few centimeters with the AMF-plant habitats positioned adjacent to each other. In the second experiment, overdispersed micro-landscapes promoted functional root colonization. This study provides experimental evidence that the spatial distribution of habitats can determine AMF abundance at the microscale.

**Supplementary information:**

The online version contains supplementary material available at 10.1007/s00572-022-01087-0.

## Introduction

Arbuscular mycorrhizal fungi (AMF) are a group of obligate symbiotic fungi that underpin many ecosystem processes (Newshan et al. 1995; van der Heijden et al. 2015). AMF form nutritional symbioses with the vast majority of terrestrial plants (Brundrett and Tedersoo 2018). The association results in several benefits to the plant host such as improved tolerance to droughts, heavy metals, and salinity but also protection of the host from pathogens which favors plant fitness (Newsham et al. 1995). At the same time, AMF alter ecosystem functioning through promoting soil aggregation, slowing nitrification and N leaching, and speeding decomposition (Nuccio et al. 2013; Leifheit et al. 2014; Powell and Rillig 2018; Veresoglou et al. 2019).

Although widespread in terrestrial ecosystems, dispersal constraints on AMF have been observed in relation to hyphal growth (i.e., Klironomos and Moutoglis 1999). The specific role of host plant distribution on AMF propagule dispersion, however, has rarely been studied. There is evidence that AMF could benefit from host plants located in proximity to each other, which has been shown experimentally at a spatial scale of a few cm (Klironomos and Moutoglis 1999). This also could be the case at larger spatial scales. In Grünfeld et al. (2019), for example, we presented evidence that root colonization rates of understory AMF hosts in temperate forests increase in the vicinity of woody species associating with AMF (Grünfeld et al. 2019). Even though, in this previous study, we did not address likely underlying mechanisms, there is a good chance that these are related to a higher availability of AMF propagules in the vicinity of trees associating with AMF (Grünfeld et al. 2019). Spatial distance between host plants thus could determine the strength of root colonization. Greater availability of AMF propagules close to host plants could also result in a denser functional (colonization consisting of arbuscules and coils) and active colonization and thus make the symbiosis more effective.

We tested these assumptions in a series of two controlled greenhouse experiments—in *experiment 1*, we manipulated the connectance between host plant patches (habitats) and hypothesized that *Medicago lupulina* roots in “micro-landscapes” showing a higher connectance would be colonized more extensively by AMF and have a higher ratio of functional structures, i.e., arbuscules and coils, to total colonization than micro-landscapes equivalent in size with a lower connectance (*experiment 1*). Higher occurrences of root mycorrhizal structures might further indicate that the plant allocates more photosynthates to the fungus which could promote the growth of exploratory AMF hyphae. We thus also hypothesized that higher root colonization of *M. lupulina* and higher ratios of functional structures would foster higher densities of extraradical hyphae in the soil connecting or surrounding the habitats.

Our “micro-landscapes” in *experiment 1* did not differ only in relation to the proximity of the four suitable AMF habitats but also in other relevant parameters such as access of the plants to soil nutrients, which should have been lower when the planted compartments were in close proximity to each other. The amount and availability of nutrients, especially phosphorus, in the soil can impede establishment and development of mycorrhizal associations. For example, plants could invest more in nutrient acquisition and transport when the soil environment is heterogeneous because environmental heterogeneity can exacerbate the energy costs to assimilate the nutrients which limit plant growth (e.g., Tsunoda et al. 2014) and can increase their mycorrhizal responsiveness (Facelli and Facelli 2002; Janos 2007). AMF may require higher rates of dispersal and root colonization in this case to achieve the same foraging success as in homogeneous landscapes. We therefore tested in a second greenhouse experiment (*experiment 2*) whether heterogeneous micro-landscapes, generated through combining unfertilized and fertilized patches of vegetated soil, would stimulate AMF root colonization, the ratio of functional structures and extraradical density between habitat patches, particularly when different habitat types were adjacent to each other more often than expected by chance. We specifically considered two treatments with mixtures of habitat, one with overdispersed and the other with aggregated micro-landscapes.

## Material and methods

### Experiment design

The two experiments were conducted in an air-conditioned greenhouse with the temperature varying between 18 and 22 °C and relative humidity between 45 and 55%. Supplementary sodium vapor lights (400 W) were programmed to run from 7:00 to 19:00 every day. *Experiment 1* was carried out between January 2018 and April 2018 and *experiment 2* between May 2018 and November 2018.

The experimental units were 90 × 90 cm boxes made of PVC-free foam (FOREX®) with a height of 20 cm which were left unvegetated. Each box contained a constant number of identical habitat patches (also referred to as vegetated inserts) each with a diameter of 8 cm and a height of 20 cm. Each insert had four pairs of 4-cm-diameter windows arranged symmetrically, covered with a 30 μm mesh which permitted access to fungal hyphae but blocked root growth. For each insert, we used 900 g of freshly collected, air dried, and sieved (1 cm sieve) natural grassland soil and added 200 seeds of *M. lupulina* at a final density of 4 seeds cm^−2^ to simulate plant individual densities in grasslands (Scotton 2019). We anticipated that insert soil contained indigenous AMF propagules at densities approximating those in the field. In contrast to the inserts, the connecting and surrounding matrix soil consisted of unvegetated, steam-sterilized soil mixed with sand at a 4:5 ratio (totalling approximately 150 kg of soil mix per mesocosm).

**Fig. 1:**
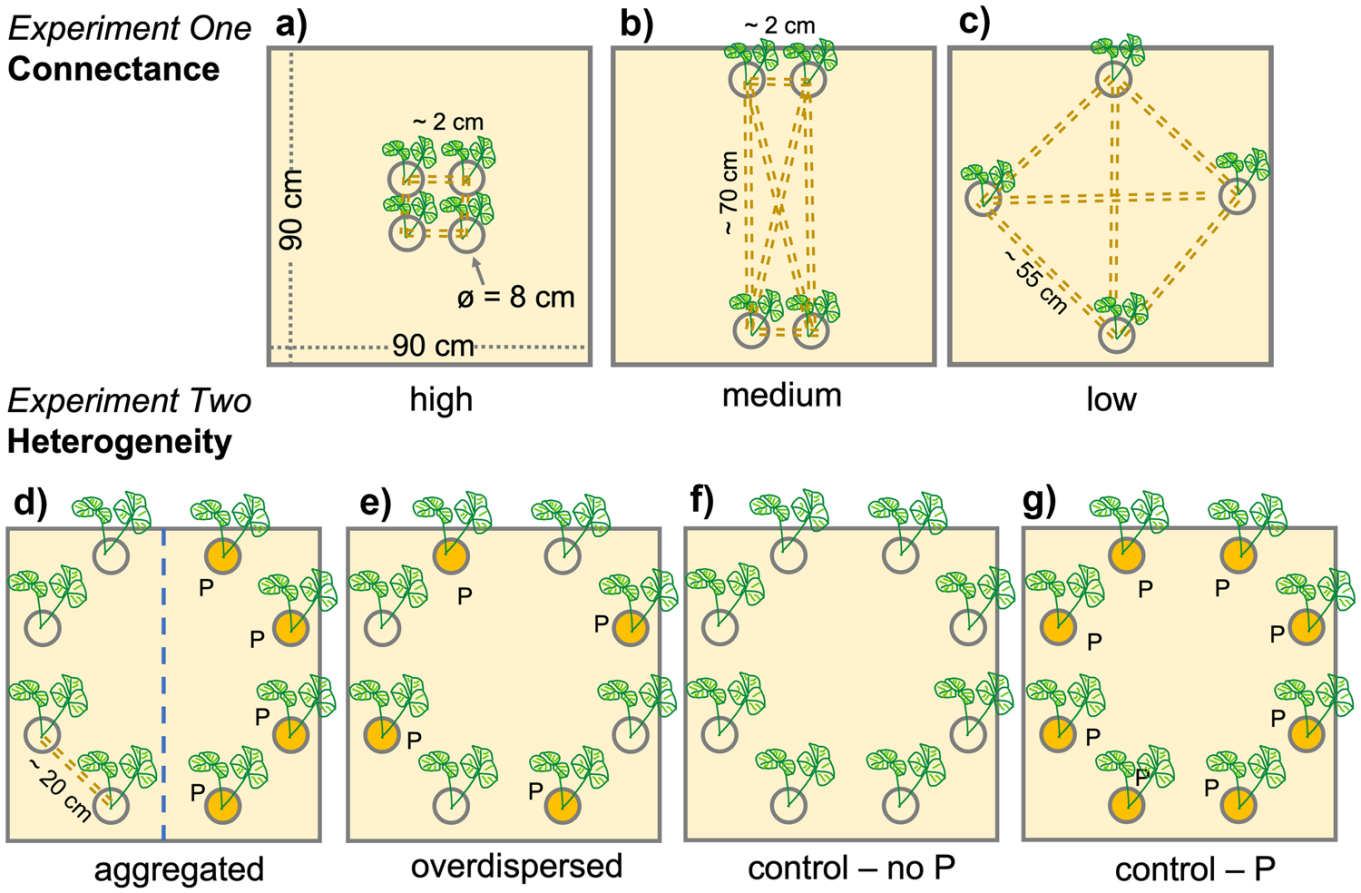
Schematic overview of the experiment setup showing the artificial micro-landscapes we generated which differed either in relation to habitat connectance (*experiment 1*) or habitat heterogeneity (*experiment 2*). Dotted lines in *experiment 1* present the minimal distances that AMF had to cross to reach another insert, which was around 2 cm in the high-connectance (**a**), 2 cm, or 70 cm in the medium-connectance (**b**), and 55 cm in the low-connectance treatment (**c**). In the second experiment, the micro-landscapes consisted of eight habitat patches differing in fertilization (yellow compartments indicate P-addition) with constant distances of 20 cm between each, arranged in four treatments, (**a**) an aggregated treatment, (**b**) an overdispersed treatment, and (**c**) two controls different in relation their fertilization state. Seeds of the host plant *Medicago lupulina* were sown at a density of 4 seeds/cm.^2^ to standardize population density among inserts

In *experiment 1*, we manipulated the spatial distribution of four inserts with the same total habitat size in three treatments: in the “high-connectance micro-landscape” (Fig. 1a), the distances between inserts were minimized; in the “medium-connectance micro-landscape” (Fig. 1b), two inserts were placed centrally on two opposite walls so that the two insert pairs faced each other with a distance of approximately 70 cm between them; and in the “low-connectance micro-landscape” (Fig. 1c), the four inserts were positioned at the corners of a schematic square with approximately 70 cm diagonals (Fig. 1c). Each treatment was replicated four times.

In *experiment 2*, we used the same boxes as in *experiment 1*, each containing eight of the above-described vegetated inserts. In this experiment, the spatial arrangement was invariable while the four treatments differed in which of the eight inserts were fertilized. For this purpose, at the beginning of the experiments, the natural grassland soil was mixed with 1.8 g of solid superphosphate in the inserts allocated to the fertilized treatment. In all treatments, the inserts were arranged as vertices of a hypothetical octagon with even distances of 20 cm between them (Fig. 1d–g). In two control treatments, we used either unfertilized (Fig. 1f) or fertilized (Fig. 1g) inserts only. In the “aggregated” and the “overdispersed” treatments, we mixed both insert types: in the aggregated treatment (Fig. 1d), the four identical inserts were positioned adjacently, forming semi-circular aggregates of either fertilized or unfertilized inserts. In the overdispersed treatment (Fig. 1e), we maximized heterogeneity by arranging the habitat types alternately, so that each fertilized insert was between two unfertilized inserts and vice versa. Each treatment was replicated four times, except for the aggregated one which was replicated six times, resulting in a total of eighteen experimental units.

### Growth settings

For both experiments, we used a fully randomized design. Because of the weight of the mesocosms, it was impossible to re-randomize the experimental units during the experiment. In both experiments, two weeks after germination of the seedlings, we set up an automatic irrigation system on a daily watering setting (over the first 2 weeks of the experiments, watering was carried out manually). We further controlled growth conditions with an Em50 data logger and five ECH_2_O EC-5 soil moisture sensors positioned in three experimental units, with one sensor in each matrix soil and in two of the three units, an additional sensor was placed in one of the inserts. Watering was adjusted so that soil moisture ranged between 60 and 75% of the water holding capacity. We inspected plant growth daily and weeded any volunteer plants from the intervening soil as well as from the inserts.

### Harvest

Following 12 weeks of growth, in both experiments, we harvested root and soil samples to assay AMF root colonization and hyphal density in the soil. Aboveground biomass was dried at 60 °C for 7 days before weighing. We took soil cores at distances of 2 cm and 10 cm from two inserts per mesocosm as well as at the center of the mesocosm, resulting in five soil samples per mesocosm. In all treatments, we focused on two inserts at opposite ends of the mesocosm and sampled along a hypothetical line between them which crossed the center. Over this hypothetical line, we measured distances of 2 and 10 cm outwards (high-connectance micro-landscape in *experiment 1*) or inwards (other treatments in both experiments) and cored the soil with a 50 ml centrifuge tube (3 cm diameter). Plant roots were stained with trypan blue and arbuscular mycorrhizal colonization was assessed using a light microscope at 200-fold magnification (McGonigle et al. 1990). Hyphae were extracted from 8 g soil using a sodium hexametaphosphate solution and were stained with trypan blue (Phillips and Hayman 1970). Soil particles including stained hyphal fragments were evenly placed on a filter paper using a vacuum pump. Hyphal density was then estimated through light microscopy at 200-fold magnification and was classified as AMF hyphae and non-AMF-hyphae based on morphological criteria. Here we report only AMF hyphae.

We additionally assayed DNA from the roots of the plants. Results of the AMF community composition in the roots in the two experiments have been described in Veresoglou et al. (2021). In that study, we observed that α- and β-diversity differed little across the micro-landscapes.

### Statistics

We tested whether root colonization changed across treatments with repeated measures ANOVAs using root colonization parameters, namely total AMF colonization and occurrence rates of arbuscules, vesicles, and coils and summed functional (coils and arbuscules) structures, respectively, as response variables. We used a spatial repeated measures approach so that we could separate variation arising from inserts belonging to the same mesocosm, termed within-subjects variation, and that observed between mesocosms, termed between-subjects variation. The name of the test can be confusing because observations usually are replicated in time and not in space and this is why the test is known as a repeated-measures ANOVA. We additionally used the ratio of occurrences of functional structures over total colonization as a response variable which potentially is indicative of the development stage of the AM symbiosis between the plant and the AMF network. Our models had as predictors the categorical variable treatment which in the case of both experiments had three levels: in *experiment 1*: low, medium, and high connectance, whereas in *experiment 2*: controls (with and without fertilization combined), overdispersed, and aggregated. The other predictor was the variable *biomass* (i.e., we corrected for differences in shoot dry weight across treatments, a continuous variable). In *experiment 2*, we additionally included a categorical parameter reflecting the habitat type describing whether there had been a fertilizer addition.

For root colonization, we assessed 100 slide intersections per sample, except for *experiment 2*, where an additional 100 intersections were assessed for two inserts per mesocosm to provide a solid database for comparing overdispersed and aggregated treatments. To compare the aggregated and overdispersed spatial designs in *experiment 2*, we carried out the analyses on the subset of observations from those two treatments, meaning that we excluded the controls, specifically targeting mesocosms where root colonization had been assessed on 200 slide intersections. Response variables were log- or square root-transformed when needed to meet the assumptions of normality and homoscedasticity.

To assess relationships between functional colonization parameters and either hyphal length or plant biomass, we used non-parametric correlation tests (Kendall’s tau statistics). We additionally used full ANOVA models (with the same structure as above) to test whether hyphal length was affected by the spatial treatments and whether hyphal length itself affected the functional ratio in both experiments. All statistical analyses were carried out in R (version 4.0.2).

## Results

### Root colonization parameters

We detected AMF root colonization in all root samples as well as AMF hyphae in the soil of all samples from both experiments. The percentage of colonized root length varied greatly between the two experiments and was overall higher in the first experiment, averaging 30%, compared to only 8% in the second experiment (Table 1). Within the colonized sections, arbuscules were the most abundant structures after hyphae. Colonization with coils and vesicles was below 3% in both experiments. The functional ratio, which indicates the proportion of functional structures, namely coils and arbuscules, over total colonization, accounted for two-thirds of total colonization in the first experiment and one-quarter in the second experiment. Phosphorus addition drastically reduced AMF root colonization in the aggregated, overdispersed, and fertilized control treatment in the target inserts (Fig. S2). Plant biomass affected total colonization in both experiments as well as arbuscular, functional colonization and the functional ratio in *experiment 1* (considering within mesocosm variability). In *experiment 1*, biomass correlated negatively with arbuscular colonization (Fig. S4a). Arbuscular colonization in contrast was highest at patches with average biomass in *experiment 2* (Fig. S4b).

**Table 1:** Percentages of total AMF root colonization and single colonization structures vesicles, arbuscules, and coils, combined for all treatments per experiment. The functional ratio indicates the proportion of functional structures (arbuscules + coils) over total colonization

AMF colonization structure	Experiment	Colonization range [%]	Median [%]	Quartiles [%]
Total colonization	*Experiment 1* *Experiment 2*	1–58 1–37	30.3 8	22.9–35.1 5–14
Vesicles	*Experiment 1* *Experiment 2*	0–7.5 0–15	2.5 1	1.5–3.5 0.5–15
Arbuscules	*Experiment 1* *Experiment 2*	0–39 0–22	17.3 1	13.5–24.8 0–2.5
Coils	*Experiment 1* *Experiment 2*	0–2.5 0–8	1 1	0.5–1.5 0–2
Functional ratio	*Experiment 1* *Experiment 2*	0–88.3 0–87.5	66.2 25	56.7–73.2 10.7–43.9

### High in connectance habitats of Medicago lupulina stimulate AMF colonization

**Fig. 2:**
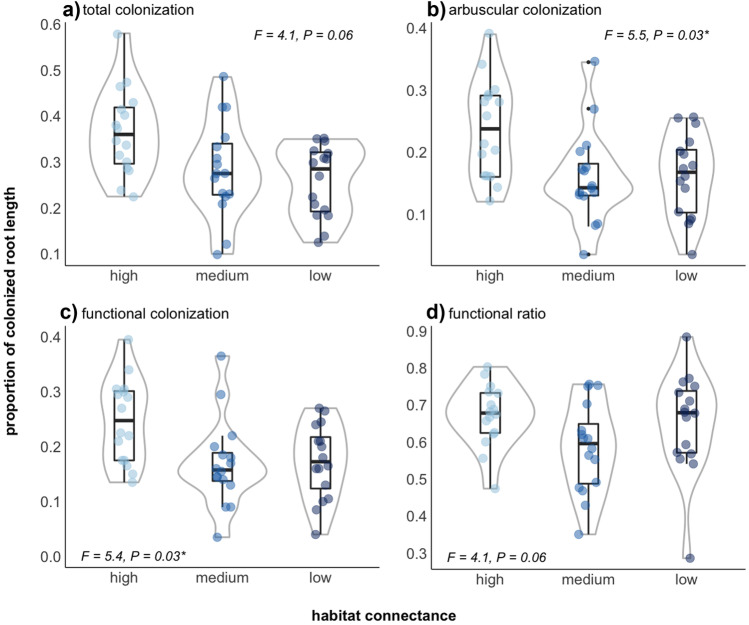
Proportion of AMF root colonization: (**a**) total and (**b**) arbuscular colonization across three levels of habitat connectance (*experiment 1*). (**c**) Functional root colonization represents the sum of arbuscules and coils per root length and (**d**) the functional ratio represents functional over total AMF colonization. Data points in light to darker blue represent the habitat connectance levels “high,” “medium,” and “low.” Four inserts (= habitat patches) within each of the four replicates resulted in *n* = 16 counts per treatment. Habitat connectance affected arbuscular and functional colonization, and marginally affected total colonization and the functional ratio (see inserted F-statistics and full ANOVA model results in Appendix II: Tests 1.1, 1.3, 1.5, and 1.6)

Arbuscular (*F*_2,8_ = 5.5, *P* = 0.03; Appendix II: Test 1.3) and functional colonization (*F*_2,8_ = 5.4, *P* = 0.03; Appendix II: Test 1.5) differed across the three connectance treatments in *experiment 1*. Since there was no effect on the formation of coils, the effects on functional colonization likely are driven entirely by changes in arbuscules. In both cases, and also for total mean colonization and the functional ratio, highest colonization values could be observed in the high-connectance micro-landscape (Fig. 2 and S1). Plant roots from the medium-connectance micro-landscape had a tendency to show lower total and functional colonization values and functional ratio (Fig. S1 and Appendix II for overall statistics).

### Heterogeneous micro-landscapes of Medicago lupulina stimulate AMF colonization

**Fig. 3:**
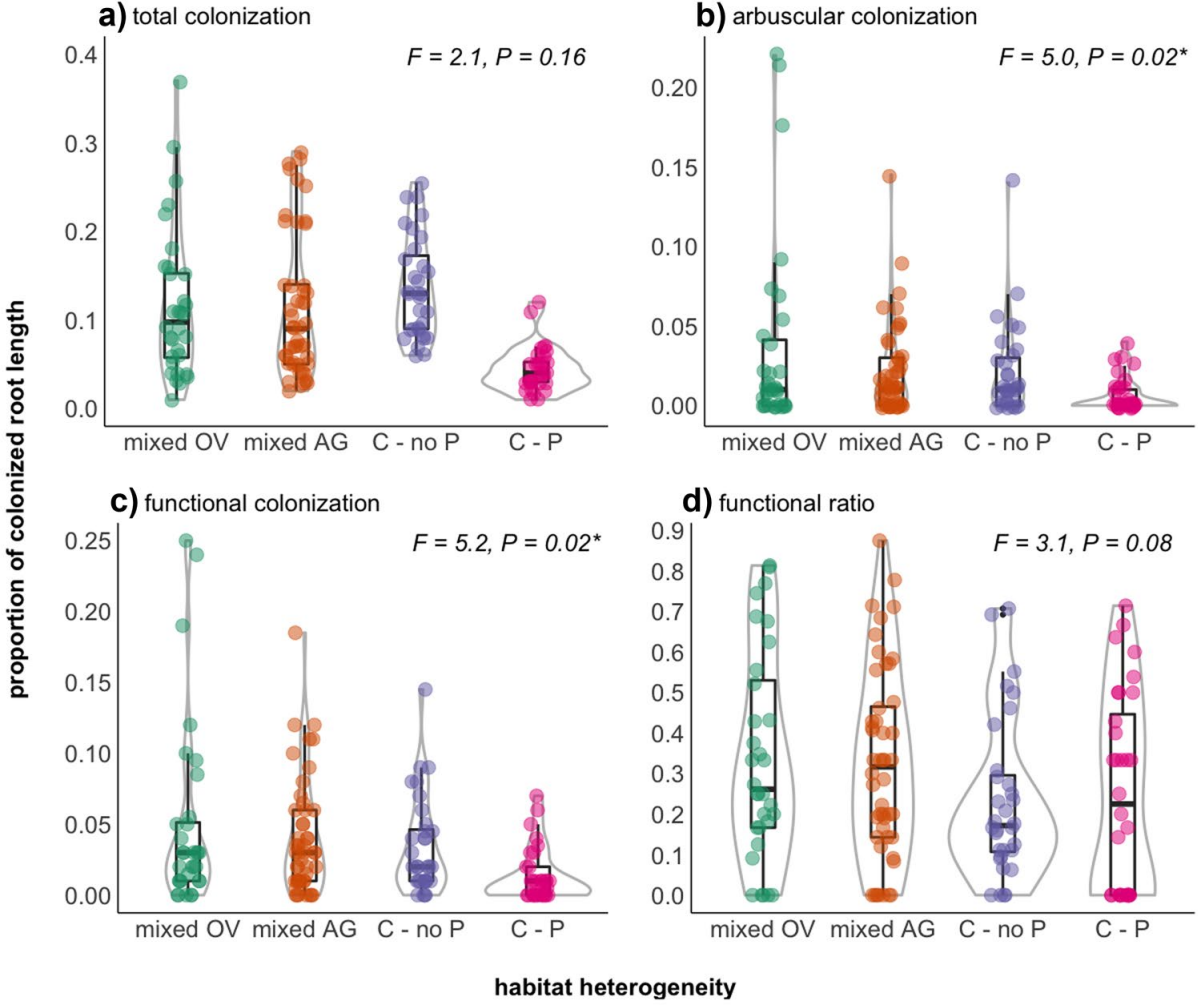
Proportion of AMF root colonization: (**a**) total and (**b**) arbuscular colonization across three levels of habitat heterogeneity (*experiment 2*). (**c**) Functional root colonization represents the sum of arbuscules and coils per root length and (**d**) the functional ratio represents functional over total AMF colonization. Green points indicate the mixed overdispersed (OV), orange the mixed aggregated (AG), purple the unfertilized control (C–no P), and pink the P-fertilized control treatment (C-P). Eight inserts (= habitat patches) within each of the four replicates resulted in *n* = 32 data points per treatment, or *n* = 48 in the case of the aggregated treatment which had six replicates. Habitat heterogeneity affected arbuscular and functional colonization (see inserted F-statistics and full ANOVA model results in the Appendix II: Tests 2.3 and 2.5). The two control levels were considered one in the ANOVA models and compared to the two mixed habitats

P-fertilization (*F*_1,13_ = 26.5, *P* = 0.0002; Fig. S2), but not the spatial treatments (*F*_2,13_ = 2.1, *P* = 0.16), reduced total AMF root colonization in *experiment 2* (Fig. 3a, Fig S2a, Appendix II: Test 2.1). We observed fewest arbuscules (*F*_2,13_ = 5.0, *P* = 0.02) in the controls and the most arbuscules in the overdispersed compared to the aggregated treatment (Fig. 3b, Fig. S2c, F_1,17_ = 5.2, *P* = 0.04; Appendix II: Test 2.3). We could not find any comparable differences for vesicles or coils (Fig. S2b, d). There was higher functional colonization (*F*_2,13_ = 5.2, *P* = 0.02; Appendix II: Test 2.5) in the heterogeneous micro-landscapes compared to controls but no clear difference in the functional ratio (Figs. 3c–d and S2e-f).

### Functional implications — extraradical hyphae

Hyphal densities did not differ across treatments of both experiments (Appendix II: Test 3.1 and Test 3.2). We further tested for relationships between extraradical hyphal density and functional colonization to test whether root colonization could have induced any exploration behavior by AMF. In the full ANOVA models, we observed an effect of mean — averaged per mesocosm — hyphal density (hyphal length in mm per g soil) on the functional ratio in *experiment 1* (*F*_1,7_ = 8.9, *P* = 0.02) but not in *experiment 2* (see Appendix II: Tests 4.1 and 4.2). In *experiment 1*, there was a trend for mean hyphal density to increase with the proportion of functional colonization (averaged by median) which we visualized in Fig. S3 (Kendall’s tau = 0.39, *P* = 0.086). We did not observe any correlation between mean hyphal density in the soil and mean functional root colonization in *experiment 2* when comparing aggregated to overdispersed micro-landscapes (Fig. S3). There was no effect of plant biomass on hyphal density in either experiment (Appendix II: Tests 3.1 and 3.2), but there was a correlation between both variables in *experiment 1* (Fig. S4, Kendall’s tau =  −0.31, *P* = 0.0017).

## Discussion

In two controlled greenhouse experiments, we observed that manipulations of the spatial distribution of combined plant, soil, and AMF habitats changed the frequencies of arbuscules. We found that habitats high in connectance (*experiment 1*) and heterogenous micro-landscapes (*experiment 2*) fostered the highest AMF root colonization in *M. lupulina*. In *experiment 1*, we found that arbuscular and functional colonization were higher in habitats high in connectance compared to medium- or low-connectance habitats of the same total habitat size. Total mean colonization showed the same tendency. In *experiment 2*, we observed that arbuscular and functional colonization (and marginally the functional ratio) were affected by the spatial treatment, showing highest colonization values in the heterogeneous treatments. This pattern was independent of the clear effects of fertilization in which P-enriched patches showed consistently low levels of root colonization. We structure this discussion so that we first address each experiment individually and later end with an overall synthesis addressing likely future perspectives arising from this study.

### High connectance habitats of Medicago lupulina stimulate AMF colonization

In *experiment 1*, we found support for our hypothesis postulating that habitats high in connectance would promote AMF root colonization and the formation of functional structures within habitat patches. We propose two possible explanations for our findings, in addition to the obvious, potential explanation that tightly packed, facultatively mycotrophic host plants diminish available P in their rhizospheres, thereby favoring elevated mycorrhiza formation.

First, we believe that *experiment 1* presents evidence that AMF associations might become more beneficial (at minimum, for the fungal associates) when the number of potential hosts increases. Larger distances between host patches might have hindered AMF dispersal and might have thus reduced the effective number of likely hosts. Because we used a relatively large amount of fresh homogenized soil for the inserts, we expected a rapid build-up of AMF mycelium and high colonization ability independent of the treatment. However, these processes could have been accelerated in the treatments in which the habitats were in the vicinity of one another and thus have given rise to our observations.

Second, we believe that these results match well with a previous study we carried out at a much larger spatial scale which showed that in forest stands with high coverage of AMF-associating trees, the roots of the herbaceous understory were colonized more extensively with AMF than in stands maintaining a low-coverage of AMF-associating trees (Grünfeld et al. 2019) even though they did not differ in AMF community composition (Grünfeld et al. 2021). Both in Grünfeld et al. (2019) and here, we observed that a relatively higher proximity to likely AMF hosts led to AMF more extensively colonizing plant hosts. In that regard, we observe a consistent pattern at two very different spatial scales on how host availability influences root colonization by Glomeromycota. Our results also show parallels to published experimental data in which root colonization was enhanced in compartments connected by common mycorrhizal networks compared to disconnected compartments (Weremijewicz and Janos 2013).

It is likely that the spatial distribution of AMF hyphae shows comparable patterns in some non-woody habitats such as agricultural landscapes and urban environments. The causes of AMF hyphal distribution, however, differ. In natural forests, the fragmentation of suitable AMF habitats happens mainly because of the heterogeneous distribution of suitable hosts. In agricultural landscapes, the causes might be related to management practices suboptimal for conserving AMF combined with different proximity of crop individuals to adjacent vegetation patches. In the case of urban areas, by contrast, it might be the patchiness of vegetation that causes dispersal limitation among AMF. Therefore, we suggest that the results of *experiment 1* are of great relevance for a better understanding of distribution patterns of AMF.

### Heterogeneous micro-landscapes of Medicago lupulina stimulate AMF colonization

In *experiment 2*, we manipulated the effective distances between different habitat types (unfertilized and P-fertilized soil) and expected that this would alter AMF root colonization patterns. We specifically hypothesized that micro-landscapes with high habitat heterogeneity would promote high colonization rates because they require high foraging investments by AMF. We indeed observed that *M. lupulina* individuals in overdispersed habitats showed higher absolute AMF colonization values than AMF in aggregated habitats (Fig. 3a and S2a). This result supports the idea that habitat heterogeneity at the micro-landscape scale, which was represented by the contrast of overdispersed vs. aggregated micro-landscapes, could be equally important for ecosystem functioning as it may be at large scale. The majority of the existing literature on landscape diversity describes studies at relatively large spatial scales (i.e., dimensions of habitats exceeded 10 m; Madritch et al. 2009; Aragón et al. 2011; Sowińska-Świerkosz and Soszynski 2014). It remains unknown if these results on habitat heterogeneity are generalizable to other systems. For example, AMF play a role in crop production; it could therefore be an interesting follow-up question whether sowing schemes in agriculture induce differences in the functioning of AMF symbioses compared to the usual patchier distribution of the hosts in natural habitats.

We believe that heterogeneity in habitat types is poorly captured across experimental studies investigating AMF root colonization or community composition. Most experiments on AMF use soil that has been pre-treated so that the existing AMF propagules are eliminated (Chaudhary et al. 2016). This process includes practices such as the sterilization of soil but often also a step of soil homogenization via sieving (as in our matrix soil). It is likely that as a result, our current understanding of arbuscular mycorrhizas is biased towards homogeneous habitats and it would be interesting to explore if and to what degree AMF colonization dynamics differ under more natural settings.

### Synthesis and future perspectives

Across two experiments, we consistently observed that the spatial distribution of *M. lupulina* vegetated habitats altered AMF abundance in the roots of the host (i.e., total root colonization, but also occurrence rates of arbuscules and coils compared to total colonization). These findings could help us synthesize past studies of AM associations in which the spatial distribution of AMF habitats varied, either intentionally or unintentionally. It is a common practice, for example, to set up experiments with multiple plant host individuals, particularly when these target plant species have a low germination ability. In such cases, it might be expected that multiple plants become colonized extensively with AMF. In *experiment 1*, despite the fact that we compared systems with multiple plant hosts, *M. lupulina* showed up to 23% additional total root colonization in the high-connectance micro-landscape compared to the low-connectance micro-landscape. The observation that the spatial distribution of AMF habitats determines the intensity of root colonization could help us plan more effective and interpretable future studies, with multiple plant individuals per experimental unit.

A key element of novelty in this study is that we showed that the spatial distribution of the inserts altered arbuscular colonization. We interpret these changes as evidence that spatial distribution of the AMF habitats alters the development of mycorrhizas. There is a lack of studies assessing relationships between distinct AMF structures and often positive correlations between extraradical hyphae and colonization rates are assumed without empirical evidence. Our results suggest that different AMF structures might be unequally distributed in the field, and that this might be related to host plant connectivity and soil heterogeneity. We could find only a weak tendency in one of the experiments for our assumption that the functional ratio (functional over total colonization) correlates positively with densities of extraradical hyphae across the mesocosms. A follow-up question of this study is whether the change in mycorrhizal dynamics eventually induces ecologically significant changes in the fitness of the host. In Veresoglou et al. (2017), we showed that in the proximity of woody hosts in temperate forests there is a higher occurrence rate (and overall diversity) of AM-associating herbaceous plants. It is quite likely that the underlying mechanism relates to a higher fitness of hosts in the vicinity of accumulations of AMF propagules (i.e., which we assume in the presence of large woody hosts) and that this mechanism actually drove the distribution patterns of herbaceous hosts in that study. It remains unclear how ubiquitous the diversity patterns that we observed in Veresoglou et al. (2017) might be, but if common, we see scope in exploring avenues to integrate concepts from meta-community theory to describe mycorrhizal dynamics (Veresoglou et al. 2012). If this is a general pattern, the integration of meta-community theory (Leibold et al. 2004), considering host plants as patches of local AMF communities connected by dispersal, could be suitable to effectively describe dynamics of symbiotic organisms such as AMF (Costello et al. 2012; Mihaljevic 2012; Veresoglou et al. 2012; Christian et al. 2015).

To conclude, we present experimental evidence that the spatial distribution of AMF habitats is important for the development of mycorrhizas. In this study, we linked rarely considered parameters, namely host connectance and soil heterogeneity, to the abundance of AMF root colonization and extraradical hyphae. It would be helpful to invest in controlled experiments with different spatial designs to better understand AMF community dynamics. Results from such studies could be used to infer mechanisms of AMF dispersal, allowing us to better understand AMF distributions in natural landscapes, which ultimately may provide an important knowledge base for developing conservation strategies for these important soil organisms.

## Supplementary information

Below is the link to the electronic supplementary material.

Supplementary file1 (PDF 888 KB)

## Data Availability

Data and code are available on request.
