# Supplementary material for: Arbuscular mycorrhizal root colonization depends on the spatial distribution of the host plants
Source: Mycorrhiza. 2022 Jul 6;32(5-6):387–95. doi: 10.1007/s00572-022-01087-0 (PMC9561028; doi:10.1007/s00572-022-01087-0)
Supplement: Supplementary file 1 — Supplementary file1 (PDF 888 KB) [file 572_2022_1087_MOESM1_ESM.pdf]

Supporting Information

Article title: Arbuscular mycorrhizal root colonization depends on the spatial structure of the host plants

Authors: Leonie Grünfeld, George Skias, Matthias C Rillig, Stavros D Veresoglou

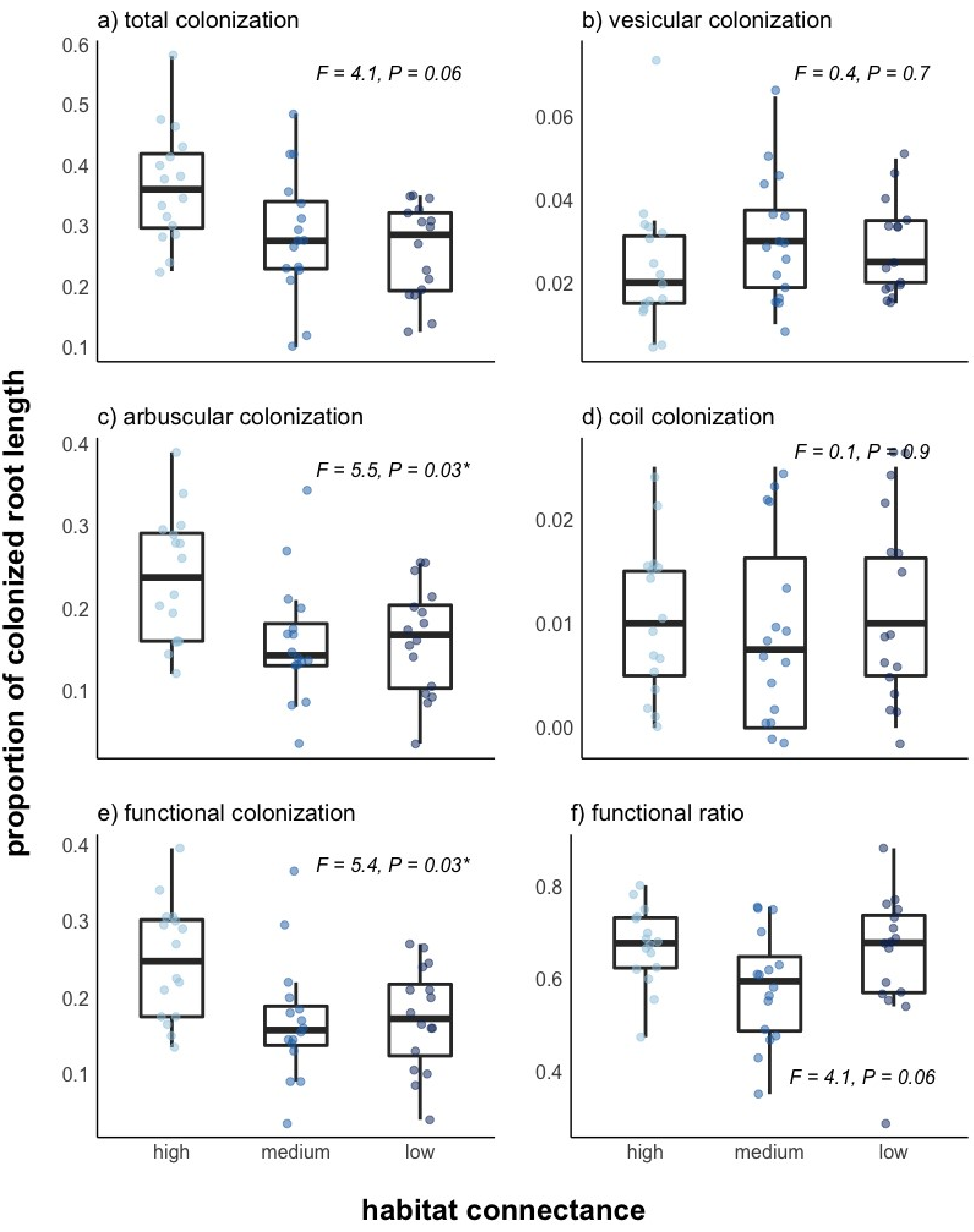

Fig. S1: Overview of the responses of the different colonization measures with habitat connectance (*Experiment One*). The panels display a) total, b) arbuscular, c) coil, d) vesicular, e) functional (additive arbuscular + coil) colonization and f) the functional colonization ratio. Data points in light to darker blue represent the connectance levels 'high', 'medium' and 'low' within mesocosms. Habitat connectance affected arbuscular, functional colonization as well as marginally total colonization and the functional ratio (see inserted F-statistics and full ANOVA model results in the Appendix II: Tests 1.1 - 1.6).

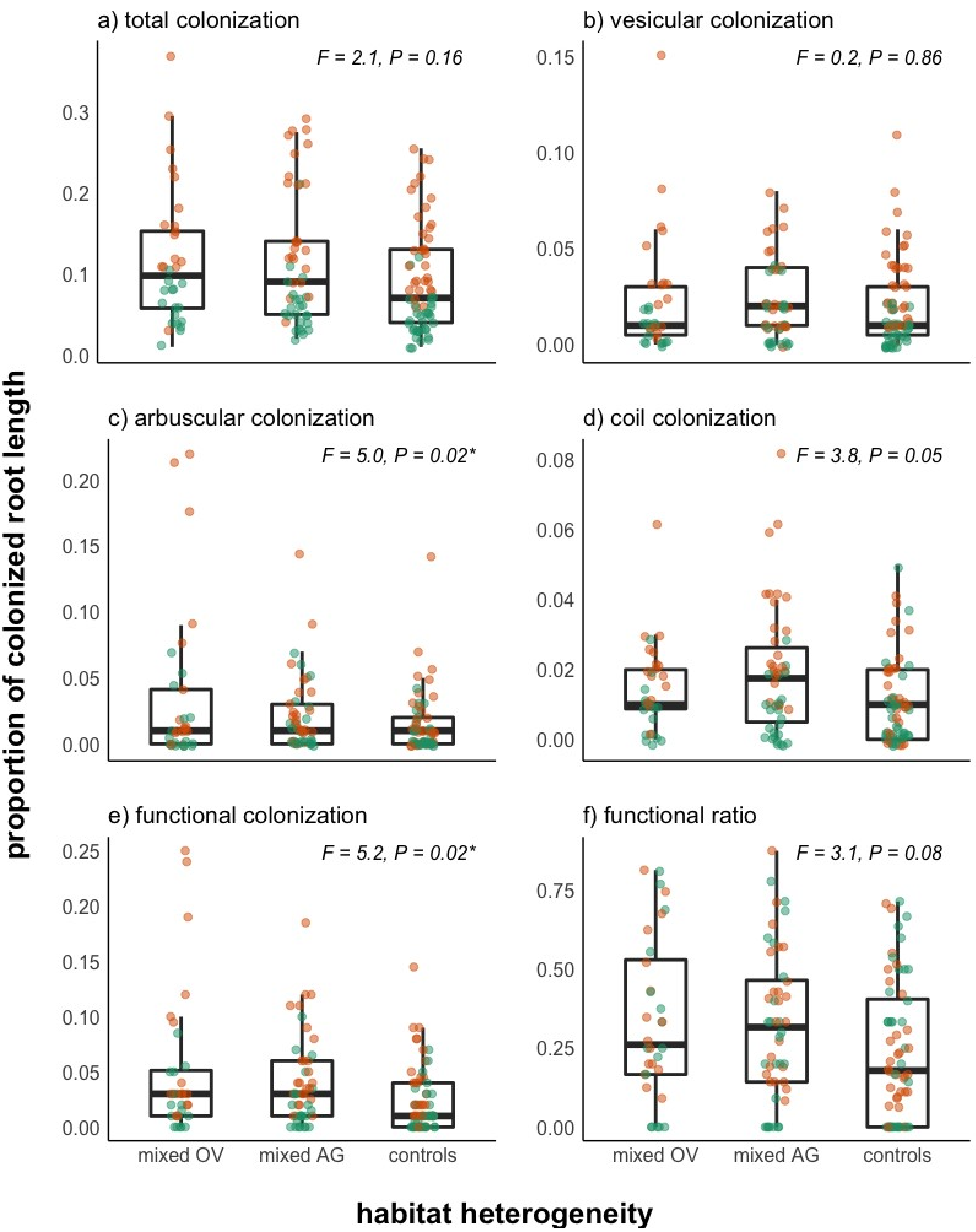

Fig. S2: Overview of the responses of different colonization measures at different levels of habitat heterogeneity (*Experiment Two*). To illustrate differences between mixed micro-landscapes and the one-habitat-type controls, the two control treatments are shown within one boxplot. The panels display a) total, b) arbuscular, c) coil, d) vesicular, e) functional (additive arbuscular + coil) colonization and f) the functional colonization ratio. Phosphorous-fertilized inserts (habitat patches) are shown in green and unfertilized ones in orange. Habitat heterogeneity affected arbuscular and functional colonization (see inserted F-statistics and full ANOVA model results in the Appendix II: Tests 2.1 - 2.6).

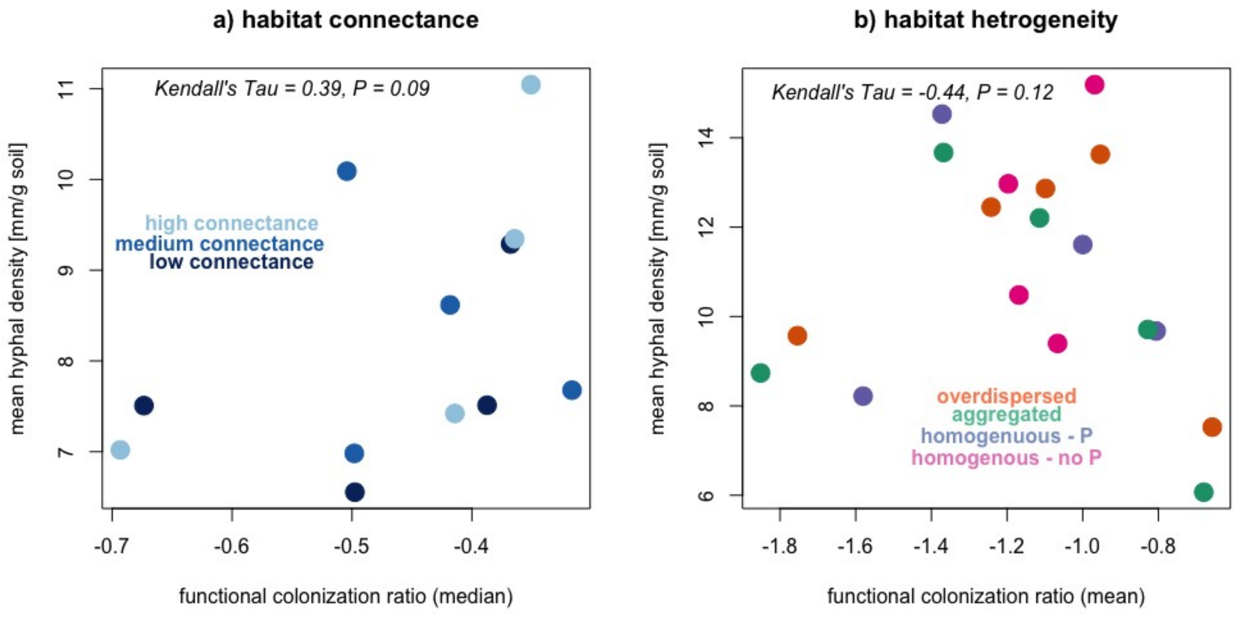

Fig. S3: Mean density of extraradical hyphae (hyphal length in mm per g soil) over functional colonization ratio for a) *Experiment One* (habitat connectance) and b) *Experiment Two* (habitat heterogeneity). Kendall's Tau statistics did not reveal any clear correlation between the mean of hyphal density and the median (a) or mean (b) of the functional colonization ratio. In the full ANOVA models, hyphal density affected the functional ratio in *Experiment One*, but not in *Experiment Two* (see Appendix II: Tests 4.1 - 4.2).

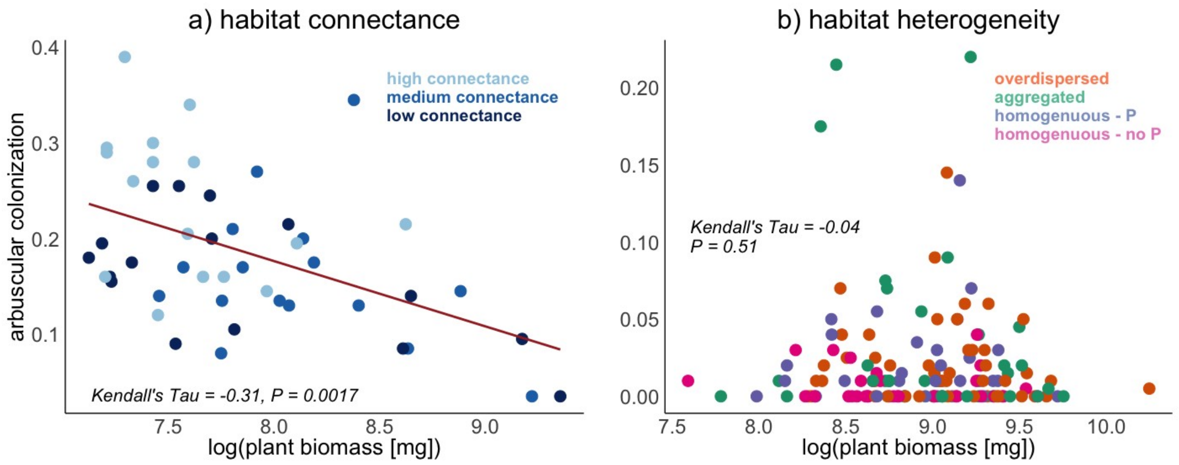

Fig. S4: Proportion of arbuscular colonization per root length and host plant biomass at the respective habitat patches. *In Experiment One* (a) arbuscular colonization was negatively correlated (Kendall's Tau, P = 0.0017) with plant biomass indicated by a linear regression line, whereas biomass affected arbuscular colonization only when considering within unit differences (F = 7.6, P = 0.009, see Appendix II for full ANOVA model results: Test 1.3). There was no effect of biomass on arbuscular colonization in *Experiment Two* (b).

Appendix I: Exploratory study & soil preparation

We used natural soil from an unmanaged grassland in Dallgow-Döberitz near Berlin (Germany, N:52.51°, E:13.14°) dominated by *Tanacetum vulgare, Daucus carota* and *Calamagrostis epigejos.* In a probe study, we grew *Plantago lanceolata* (i.e bait plant) over four weeks to assess the richness of the indigenous AMF community in its roots in four soils via terminal restriction fragment length polymorphism (*t*-RFLP). To assay AMF community structure, we extracted DNA from 30 µg of freeze-dried root material with the PowerSoil DNA isolation kit (MoBio Laboratories Inc., Carlsbad, CA, USA) and amplified it with the NS31-AML2 primers (Lee et al. 2008, Liu et al., 2011) to which we had adapted (both primers) fluorescent tags and carried out independent restriction digests with two restriction enzymes (i.e. HinfI and Hin1II (NlaIII) - under recommended restriction settings). Terminal peaks of purified amplicons (NucleoSpin® Gel and PCR Clean-up, Macherey-Nagel, Düren, Germany) were assayed on an ABI sequencer and were analysed for indigenous AMF richness. We collected fresh soil (with mean values of 0.13 % nitrogen, 1.75 % carbon and a pH of 6.7) from the site prior to each of the experiments. In each case, we sieved the soil with a semi-automatic sieving machine with a 1 cm mesh size to homogenize it and remove rock material. To eliminate AM fungal propagules from the unvegetated compartments, the soil was steam-sterilized at 60°C shortly before the experiment. We subsequently diluted the natural soil with sandbox sand in a proportion of 5:4 (soil:sand) for the unvegetated compartments. For the inserts we used undiluted unsterilized soil.

Appendix II: Model statistics

Abbreviations:
amf = total arbuscular mycorrhizal colonization
vesicles = vesicular colonization
arbuscules = arbuscular colonization
coil = coil colonization
func = functional colonization (coils + arbuscules)
ratio = functional colonization ratio (functional over total colonization)
treatment = Spatial arrangement of host patches
biomass = aboveground dry biomass
hyphae = hyphal density

Test 1.1: Exp1 - Total colonization

```
> summary(aov(amf~treatment+biomass+Error(factor(mesocosm)),data=roots2))

Error: factor(mesocosm)
              Df  Sum Sq Mean Sq F value Pr(>F)
treatment   2 0.09900 0.04950   4.110 0.0592 .
biomass     1 0.00875 0.00875   0.727 0.4187
Residuals   8 0.09636 0.01204

Error: Within
              Df  Sum Sq Mean Sq F value Pr(>F)
biomass     1 0.04088 0.04088   5.995 0.0195 *
Residuals  35 0.23865 0.00682
```

Test 1.2: Exp1 - Vesicular colonization

```
> summary(aov(vesicles~treatment+biomass+Error(factor(mesocosm)),data=roots2))

Error: factor(mesocosm)
                 Df    Sum Sq   Mean Sq F value Pr(>F)
treatment      2 0.0002885 0.0001443   0.375  0.699
biomass        1 0.0003989 0.0003989   1.036  0.339
Residuals      8 0.0030808 0.0003851

Error: Within
          Df   Sum Sq   Mean Sq F value Pr(>F)
biomass    1 0.000179 0.0001788   1.069  0.308
Residuals 35 0.005852 0.0001672
```

Test 1.3: Exp1 - Arbuscular colonization

```
> summary(aov(arbuscules~treatment+biomass+Error(factor(mesocosm)),data=roots2))

Error: factor(mesocosm)
          Df  Sum Sq  Mean Sq F value Pr(>F)
treatment  2 0.06255 0.031277   5.516 0.0312 *
biomass    1 0.00959 0.009589   1.691 0.2297
Residuals  8 0.04536 0.005670

Error: Within
                Df  Sum Sq  Mean Sq F value Pr(>F)
biomass    1 0.03125 0.031250   7.144 0.0113 *
Residuals     35 0.15311 0.004374
```

Test 1.4: Exp1 - Coil colonization

```
> summary(aov(coils~treatment+biomass+Error(factor(mesocosm)),data=roots2))
```

```
Error: factor(mesocosm)
          Df    Sum Sq   Mean Sq F value Pr(>F)
treatment  2 0.0000292 1.458e-05   0.112  0.895
biomass    1 0.0001287 1.287e-04   0.990  0.349
Residuals  8 0.0010400 1.300e-04

Error: Within
                Df   Sum Sq   Mean Sq F value Pr(>F)
biomass    1 3.96e-05 3.962e-05   0.657  0.423
Residuals 35 2.11e-03 6.030e-05
```

Test 1.5: Exp1 - Functional colonization

```
> summary(aov(func~treatment+biomass+Error(factor(mesocosm)),data=roots2))

Error: factor(mesocosm)
          Df  Sum Sq  Mean Sq F value Pr(>F)
treatment   2 0.06213 0.031065   5.432 0.0323 *
biomass     1 0.01194 0.011940   2.088 0.1865
Residuals   8 0.04575 0.005719

Error: Within
          Df  Sum Sq Mean Sq F value   Pr(>F)
biomass    1 0.03352 0.03352   7.502 0.00963 **
Residuals 35 0.15635 0.00447
```

Test 1.6: Exp1 - Functional colonization ratio

```
> summary(aov(ratio~treatment+biomass+Error(factor(mesocosm)),data=roots2))

Error: factor(mesocosm)
          Df  Sum Sq Mean Sq F value Pr(>F)
treatment   2 0.06792 0.03396   4.069 0.0604 .
biomass     1 0.05029 0.05029   6.026 0.0396 *
Residuals   8 0.06676 0.00835

Error: Within
          Df Sum Sq Mean Sq F value   Pr(>F)
biomass    1 0.1128 0.11283   10.63 0.00248 **
Residuals 35 0.3714 0.01061
```

Test 2.1: Exp2 - Total colonization

```
>
summary(aov(amf~treatment+fertilizer+biomass+Error(as.factor(mesocosm)),data=roots3
))

Error: as.factor(mesocosm)
           Df  Sum Sq Mean Sq F value   Pr(>F)
treatment   2 0.02120 0.01060   2.086 0.163765
fertilizer  1 0.13460 0.13460  26.496 0.000188 ***
biomass     1 0.00013 0.00013   0.026 0.875026
Residuals  13 0.06604 0.00508

Error: Within
            Df Sum Sq Mean Sq F value   Pr(>F)
fertilizer   1 0.2338 0.23382  85.010 9.7e-16 ***
biomass      1 0.0018 0.00183   0.665   0.416
Residuals  124 0.3411 0.00275
```

Aggregated vs. overdispersed

```
> summary(a<-aov(amf~factor(treatment)+fertilizer, data=roots3))
                  Df  Sum Sq Mean Sq F value  Pr(>F)
factor(treatment)  1 0.00800 0.00800   1.851 0.19142
fertilizer         1 0.06441 0.06441  14.898 0.00126 **
Residuals         17 0.07350 0.00432
```

Test 2.2: Exp2 - Vesicular colonization

```
> summary(aov(vesicles~treatment+fertilizer+biomass+Error(as.factor(mesocosm)),
data=roots3))

Error: as.factor(mesocosm)
           Df   Sum Sq  Mean Sq F value   Pr(>F)
treatment   2 0.000171 0.000086   0.154 0.858741
fertilizer  1 0.011691 0.011691  21.063 0.000507 ***
biomass     1 0.000000 0.000000   0.000 0.982632
Residuals  13 0.007216 0.000555

Error: Within
            Df  Sum Sq  Mean Sq F value   Pr(>F)
fertilizer   1 0.01128 0.011281  28.046 5.21e-07 ***
biomass      1 0.00005 0.000050   0.126    0.724
Residuals  124 0.04988 0.000402
```

Aggregated vs. overdispersed

```
> summary(a<-aov(vesicles~factor(treatment)+fertilizer, data=roots3))
                  Df    Sum Sq   Mean Sq F value Pr(>F)
factor(treatment)  1 0.0000408 0.0000408   0.242 0.6294
fertilizer         1 0.0012800 0.0012800   7.571 0.0136 *
Residuals         17 0.0028742 0.0001691
```

Test 2.3: Exp2 - Arbuscular colonization

```
> summary(aov(arbuscules~treatment+fertilizer+biomass+Error(as.factor(mesocosm)),
data=roots3))

Error: as.factor(mesocosm)
           Df   Sum Sq  Mean Sq F value Pr(>F)
treatment   2 0.009991 0.004996   5.043 0.0239 *
fertilizer  1 0.003235 0.003235   3.266 0.0939 .
biomass     1 0.000384 0.000384   0.388 0.5443
Residuals  13 0.012876 0.000990

Error: Within
            Df  Sum Sq  Mean Sq F value   Pr(>F)
fertilizer   1 0.01431 0.014311  11.688 0.000852 ***
biomass      1 0.00003 0.000028   0.023 0.879020
Residuals  124 0.15183 0.001224
```

Aggregated vs. overdispersed

```
> summary(a<-aov(sqrt(arbuscules)~factor(treatment)+fertilizer, data=roots3))
                  Df  Sum Sq Mean Sq  F value Pr(>F)
factor(treatment)  1 0.05596 0.05596   5.238 0.0352 *
fertilizer         1 0.03935 0.03935   3.682 0.0719 .
Residuals         17 0.18164 0.01068
```

Test 2.4: Exp2 - Coil colonization

```
> summary(aov(coils~treatment+fertilizer+biomass+Error(as.factor(mesocosm)),
data=roots3))

Error: as.factor(mesocosm)
           Df   Sum Sq   Mean Sq F value Pr(>F)
treatment   2 0.001878 0.0009391   3.805  0.050 .
fertilizer  1 0.000657 0.0006566   2.661  0.127
biomass     1 0.000033 0.0000333   0.135  0.719
Residuals  13 0.003208 0.0002468

Error: Within
            Df   Sum Sq  Mean Sq F value   Pr(>F)
fertilizer   1 0.006661 0.006661  45.188 5.83e-10 ***
biomass      1 0.000181 0.000181   1.231    0.269
Residuals  124 0.018279 0.000147
```

Aggregated vs. overdispersed

```
> summary(a<-aov(sqrt(coils)~factor(treatment)+fertilizer, data=roots3))
                  Df  Sum Sq  Mean Sq F value  Pr(>F)
factor(treatment)  1 0.00206 0.002057   0.998 0.33170
fertilizer         1 0.02725 0.027250  13.224 0.00204 **
Residuals         17 0.03503 0.002061
```

Test 2.5: Exp2 - Functional colonization

```
> summary(aov(func~treatment+fertilizer+biomass+Error(as.factor(mesocosm)),
data=roots3))

Error: as.factor(mesocosm)
           Df   Sum Sq  Mean Sq F value Pr(>F)
treatment   2 0.015738 0.007869   5.200 0.0219 *
fertilizer  1 0.006806 0.006806   4.498 0.0537 .
biomass     1 0.000644 0.000644   0.425 0.5257
Residuals  13 0.019672 0.001513

Error: Within
            Df  Sum Sq Mean Sq F value   Pr(>F)
fertilizer   1 0.04050 0.04050  25.514 1.53e-06 ***
biomass      1 0.00035 0.00035   0.223    0.638
Residuals  124 0.19683 0.00159
```

Aggregated vs. overdispersed

```
> summary(a<-aov(sqrt(func)~factor(treatment)+fertilizer, data=data2))
                  Df  Sum Sq Mean Sq F value Pr(>F)
factor(treatment)  1 0.03278 0.03278   3.870 0.0657 .
fertilizer         1 0.06381 0.06381   7.532 0.0138 *
Residuals         17 0.14401 0.00847
```

Test 2.6: Exp2 - Functional colonization ratio

```
>
summary(aov(ratio~treatment+fertilizer+biomass+Error(as.factor(mesocosm)),data=root
s3))

Error: as.factor(mesocosm)
           Df Sum Sq Mean Sq F value Pr(>F)
treatment   2 0.2909 0.14543   3.106  0.079 .
fertilizer  1 0.0037 0.00367   0.078  0.784
biomass     1 0.1754 0.17536   3.745  0.075 .
Residuals  13 0.6087 0.04683

Error: Within
            Df Sum Sq Mean Sq F value Pr(>F)
fertilizer   1  0.131 0.13056   2.419  0.122
biomass      1  0.065 0.06547   1.213  0.273
Residuals  124  6.693 0.05398
```

Aggregated vs. overdispersed

```
> summary(a<-aov(ratio~factor(treatment)+fertilizer, data=data2))
      Df Sum Sq Mean Sq F value Pr(>F)
factor(treatment)  1 0.1308 0.13082   2.876  0.108
fertilizer         1 0.0022 0.00221   0.049  0.828
Residuals         17 0.7732 0.04548
```

Test 3.1 Exp 1: Hyphal density

```
> summary(aov(hyphae ~  biomass + treatment  + Error(as.factor(mesocosm)),
data=roots2))

Error: as.factor(mesocosm)
          Df Sum Sq Mean Sq F value Pr(>F)
biomass    1   3.23   3.231   0.336  0.578
treatment  2   6.80   3.399   0.354  0.713
Residuals  8  76.88   9.610
```

```
Error: Within
          Df    Sum Sq   Mean Sq F value Pr(>F)
biomass    1 7.500e-31 7.484e-31   0.451  0.506
Residuals 35 5.804e-29 1.658e-30
```

Test 3.2 - Exp 2: Hyphal density

```
> summary(aov(hyphae ~  biomass + treatment  + Error(as.factor(mesocosm)),
data=roots3))

Error: as.factor(mesocosm)
          Df Sum Sq Mean Sq F value Pr(>F)
biomass    1   58.7   58.74   1.020  0.330
treatment  2   37.8   18.89   0.328  0.726
Residuals 14  806.4   57.60

Error: Within
           Df    Sum Sq   Mean Sq F value Pr(>F)
biomass     1 1.870e-28 1.866e-28   1.182  0.279
Residuals 125 1.974e-26 1.579e-28
```

Test 4.1 – Exp 1: Functional ratio accounting for hyphal density

```
> summary(aov(ratio ~ hyphae + treatment + biomass +
Error(factor(mesocosm)),data=roots2))

Error: factor(mesocosm)
          Df  Sum Sq Mean Sq F value  Pr(>F)
hyphae     1 0.03330 0.03330   8.872 0.02056 *
treatment  2 0.07842 0.03921  10.446 0.00792 **
biomass    1 0.04698 0.04698  12.516 0.00950 **
Residuals  7 0.02627 0.00375

Error: Within
          Df Sum Sq Mean Sq F value  Pr(>F)
biomass    1 0.1617 0.16173   17.55 0.00018 ***
Residuals 35 0.3225 0.00921
```

Test 4.2 – Exp 2: Functional ratio accounting for hyphal density

```
> summary(aov(ratio ~ hyphae + biomass + treatment + fertilizer +
Error(as.factor(mesocosm)),data=roots3))

Error: as.factor(mesocosm)
           Df Sum Sq Mean Sq F value Pr(>F)
hyphae      1 0.0147 0.01466   0.303 0.5918
biomass     1 0.0328 0.03282   0.679 0.4259
treatment   2 0.4504 0.22519   4.662 0.0318 *
fertilizer  1 0.0012 0.00118   0.024 0.8784
Residuals  12 0.5796 0.04830

Error: Within
            Df Sum Sq Mean Sq F value Pr(>F)
biomass      1  0.006 0.00638   0.118 0.7320
fertilizer   1  0.168 0.16810   3.104 0.0805 .
Residuals  124  6.714 0.05415
```
